# Real-Time On-Site Monitoring of Viruses in Wastewater Using Nanotrap^®^ Particles and RICCA Technologies

**DOI:** 10.3390/bios14030115

**Published:** 2024-02-21

**Authors:** Vishnu Sharma, Hitomi Takamura, Manish Biyani, Ryo Honda

**Affiliations:** 1 BioSeeds Corporation, Ishikawa Create Labo-202, Asahidai 2-13, Nomi 923-1211, Ishikawa, Japan; vishnusharma@biyanicolleges.org; 2 Faculty of Geosciences and Civil Engineering, Kanazawa University, Kanazawa 920-1164, Ishikawa, Japan; shimu.811@gmail.com (H.T.); rhonda@se.kanazawa-u.ac.jp (R.H.)

**Keywords:** wastewater-based epidemiology (WBE), virus, SARS-CoV-2, Nanotrap magnetic virus particles, isothermal nucleic acid amplification

## Abstract

Wastewater-based epidemiology (WBE) is an effective and efficient tool for the early detection of infectious disease outbreaks in a community. However, currently available methods are laborious, costly, and time-consuming due to the low concentration of viruses and the presence of matrix chemicals in wastewater that may interfere with molecular analyses. In the present study, we designed a highly sensitive “Quick Poop (wastewater with fecal waste) Sensor” (termed, QPsor) using a joint approach of Nanotrap microbiome particles and RICCA (RNA Isothermal Co-Assisted and Coupled Amplification). Using QPsor, the WBE study showed a strong correlation with standard PEG concentrations and the qPCR technique. Using a closed format for a paper-based lateral flow assay, we were able to demonstrate the potential of our assay as a real-time, point-of-care test by detecting the heat-inactivated SARS-CoV-2 virus in wastewater at concentrations of 100 copies/mL and within one hour. As a proof-of-concept demonstration, we analyzed the presence of viral RNA of the SARS-CoV-2 virus and PMMoV in raw wastewater samples from wastewater treatment plants on-site and within 60 min. The results show that the QPsor method can be an effective tool for disease outbreak detection by combining an AI-enabled case detection model with real-time on-site viral RNA extraction and amplification, especially in the absence of intensive clinical laboratory facilities. The lab-free, lab-quality test capabilities of QPsor for viral prevalence and transmission in the community can contribute to the efficient management of pandemic situations.

## 1. Introduction

SARS-CoV-2 is one of the most misfortunate epidemics of the 21st century. It has spread all over the world and caused respiratory infections with two distinct phases: a viral response phase and a chronic inflammation phase [1,2]. The severity of SARS-CoV-2 has been monitored to adjust infection control policies appropriately [3]. Usually, the disease severity of SARS-CoV-2 was centered on tracking the reported cases including new cases, patients admitted to hospitals, and fatalities. In the meantime, most COVID-19 surveillance is based on individual diagnostic testing, such as nucleic acid amplification assays and rapid antigen tests [4]. With these available testing strategies, several drawbacks have emerged; for example, they provide an incorrect prevalence of the true condition due to a lack of data for cases with mild or no symptoms or due to the limited diagnostic tests [5,6]. This bottleneck contributes to the proliferation of many undiagnosed cases, particularly during epidemic peaks or in regions with limited resources. The ineffective clinical surveillance systems are confronted with the unique challenge of latency periods between SARS-CoV-2 exposure, symptom onset, and the identification of cases, hospitalizations, or fatalities [7,8,9]. In addition, frequent diagnostic testing for the whole population is also burdened with an exorbitant cost, with a typical COVID-19 PCR test costing a minimum between USD 1.29 and 4.30 per test and an antigen test costing USD 5 per test [10].

Recently, wastewater-based epidemiology (WBE) has gained importance as a sensitive, non-invasive, quick, and cost-effective monitoring strategy for identifying the distribution of COVID-19 cases in a wastewater catchment [3,4,10]. Wastewater epidemiology enables the tracking of virus molecules from symptomatic- to asymptomatic-infected individuals [11,12] without the presence of overt disease manifestations [13,14,15]. The root explanation is the viral introduction process, in which, before symptoms develop, virus molecules regularly move via residential and communal sewer system pipes, in the form of excreta and body fluids (e.g., saliva and nasal secretions), to the community’s local wastewater catchments and enrich the raw sewage with viral gene fragments [16,17,18]. Hence, monitoring the virally enriched untreated wastewater at a wastewater treatment plant (WWTP) enables the identification of an increasing/decreasing trend and size of infected population in a community of the sewer catchment [19,20]. Moreover, WBE can target a smaller community by monitoring a manhole or a building unit for early detection of group infection in a care facility, dormitory, etc., where subsequent individual clinical testings enable early identification and quarantine of the infected individuals [15,21,22].

Researchers have used a broad range of concentration and amplification methods, followed by multiple infectious mathematical models and artificial intelligence in wastewater epidemiology to assess the prevalence of COVID-19 or anticipate subsequent outbreaks in a catchment [6,21,23,24,25,26,27,28,29]. Despite this, previous studies have highlighted various limitations, such as the viral recovery method, the sample source and volume, the absence of a nucleic acid extraction standard, and the limited primer and PCR testing kits [30,31,32,33,34,35]. Methods such as Polyethylene glycol (PEG) precipitation, skim milk flocculation, ultracentrifugation, and ultrafiltration are frequently utilized for concentrating SARS-CoV-2 in wastewater [36,37,38,39], and sophisticated laboratory conditions or equipment and specialized professionals are necessary for the quantitative monitoring of viruses in wastewater [40,41,42,43].

We developed the “QPsor” (Quick Poop Sensor), a very sensitive method for the identification of SARS-CoV-2 RNA in wastewater. QPsor involves the concentration of viral particles, followed by quick RNA extraction and rapid amplification steps from the wastewater samples. It primarily utilizes Nanotrap microbiome particles, which are highly porous hydrogel particles. These particles capture and concentrate contaminants like viruses, bacteria, and live infectious pathogens from complex biological mixtures including blood, saliva, urine, and wastewater. The particles also help to separate contaminants from interfering components, capture numerous target molecules from complicated metrics of one sample, and preserve target molecules from degradation [37,44].

In addition, to increase the efficiency of the Nanotrap microbiome particles, we used a cellulose–acetate filter during sampling to undertake cold sterilization of the wastewater medium for the absolute elimination of yeasts and molds, but not viruses [45]. Next, the direct amplification of viral lysates from Nanotrap microbiome particles utilizing RICCA (RNA Isothermal Co-assisted and Coupled Amplification), an ultra-sensitive and ultrarapid, one-pot isothermal nucleic acid amplification, allowed us to achieve lab-free robustness [46]. Furthermore, we also report that following a qualitative analysis of SARS-CoV-2 in wastewater by RICCA, lab-free quantification of viral RNA can be performed by applying viral lysates from Nanotrap microbiome particles on PicoGene PCR1100 (Nippon Sheet Glass, Tokyo, Japan), an improved alternative microfluidic mobile qPCR [47].

For these reasons, it is necessary to establish an efficient and prompt monitoring approach that quantifies viral RNA in wastewater in low-prevalence settings that is on-site-applicable, considers viral dissemination, and is validated with statistical data to predict COVID-19 cases.

## 2. Materials and Methods

### 2.1. Sample Collection

Wastewater samples were taken from November 2022 to March 2023 during the 8th wave of the COVID-19 outbreak from the community manholes and influent lines of wastewater treatment plants in the Ishikawa prefecture, Japan. All the samples were collected in sterilized bottles and transported at a temperature of 4 °C to the laboratory. Until further analysis, the samples were kept at −80 °C. All optimization research was performed in the BSL-2+ laboratory. To further verify the effectiveness of the optimized Qpsor, real-time testing was also performed at the on-site wastewater treatment facility center in the Ishikawa prefecture of Japan.

### 2.2. Sample Preparation

Prior to the concentration procedure, samples were centrifuged/filtered to remove suspended particles. The filtration protocol was carried out using a cellulose–acetate membrane-attached syringe filtration system (Advantec^®^ MFS, Inc., Dublin, CA, USA). This flow-through was utilized in the concentration step. The samples were centrifuged for 10 min at 3000× *g* and 4 °C, and the supernatant was utilized in the subsequent concentration procedures. The residues of suspended particles were discarded. In the context of the positive control, three different concentrations of normal saline and wastewater samples inoculated with the heat-inactivated SARS-CoV-2 variant B.1.1.7 (ATCC^®^ VR-3326HK^TM^, San Diego, CA, USA) were standardized. Using serial dilution for 100 mL volume, 1 × 10^4^, and 10^2^ copy/mL concentration, seeded samples were established and homogenized with overnight stirring at 4 °C. Prior to processing, only an aliquot of 50 mL was handled for viral concentration.

### 2.3. Virus Concentration

**Figure 1:**
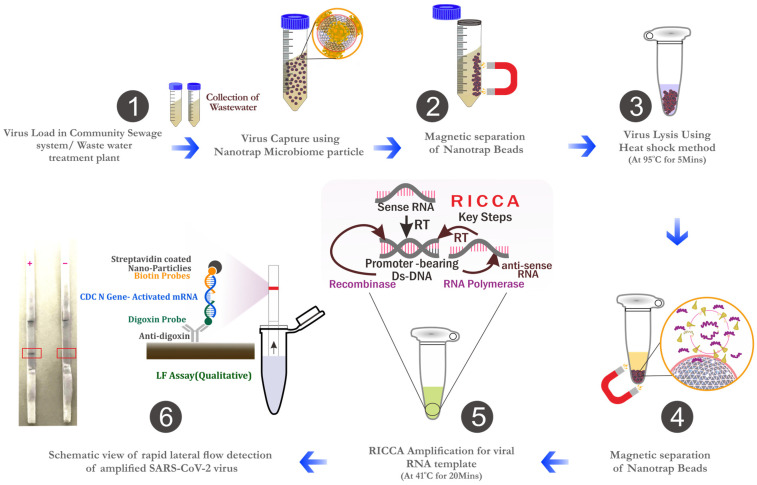
A schematic layout of the QPsor system to monitor SARS-CoV-2 viruses in wastewater. Possible results (test line positive or negative) are highlighted by red box in step-6.

The concentration of SARS-CoV-2 was evaluated using two methods: (a) the Nanotrap magnetic virus particles (NMVP) method and (b) the PEG precipitation method [37,48,49]. To evaluate each concentration stage, a pre-treated aliquot of 50 mL was divided into two additional aliquots of 10 mL and 40 mL, each of which was then processed respectively. All experiments were conducted twice using the same sample matrix (Figure 1).

The NMVP method used Nanotrap microbiome-A particles (SKU 44202-30, Ceres Nanosciences Inc., Manassas, VA, USA), which are magnetically functionalized affinity catch hydrogel nanoparticles that capture and concentrate biologically active microparticles [29,37]. In this procedure, 10 mL of the pre-treated aliquot in a sterile 15 mL conical falcon tube (Iwaki, Japan) was mixed with 100 µL of Nanotrap enhancement reagent-2 (ER2) (Ceres Nanoscience’s SKU 10112-10) and gently shaken for 10 s. After that, 150 µL of Nanotrap microbiome particles (Ceres Nanoscience’s SKU 44202-30) was added to the sample and mixed via gentle shaking. After inverting the tubes two or three times to ensure optimum mixing, the mixture was incubated at room temperature for 10 min. Then, tubes were subsequently positioned on a magnetic rack (IBA Lifesciences GmbH, Göttingen, Germany) to pelletize the Nanotrap microbiome particles at the tube’s bottom. The supernatant was discarded without disturbing the magnetic virus particle pellet and resuspended by adding 500 µL of molecular-grade water. It was transferred to a sterile 1.5 mL microcentrifuge tube and placed on a magnetic rack (MagneSphere^®^ Technology Magnetic Separation Stands, Promega (Madison, WI, USA)) to produce a final Nanotrap magnetic virus particle pellet. The resulting virus particle pellet was utilized for nucleic acid extraction. Progressively, to improve the sensitivity of virus concentration via Nanotrap microbiome particles, we increased the volume of the pre-treated sample from 10 mL to 30 mL, while maintaining the same optimum conditions.

In PEG precipitation method, viral particles in a sample were concentrated according to Alamin et al. 2022 [50]. Briefly, 40 mL of the sample was centrifuged at 5000× *g* for 5 min to eliminate large particles, then mixed with 4 g of PEG 8000 and 2.3 g of NaCl to the final concentrations of 10% PEG and 1 M NaCl. After overnight homogenization at 4 °C to equilibrate, the mixture was then centrifuged for 30 min at 10,000× *g*. Then, the pellet was suspended in 500 µL of phosphate buffer, while the supernatant was discarded.

### 2.4. Nucleic Acid Extraction

Viral RNA was extracted from both concentrated samples separately using/modifying the procedures recommended by the technology manufacturers. For Nanotrap microbiome particle separation, nucleic acids were extracted from the resultant pellet using heat shock treatment, using the modification in the procedure outlined in Ceres Nanoscience’s APP-034. Briefly, each pellet was treated with 7 µL of 1 × PBS (Phosphate-buffered saline) and 23 µL of Buffer AVE (QIAamp viral elution buffer) from the QIAamp Viral RNA Mini Kit. After 30 s of vigorous vortexing, the NMVP pellet was thoroughly incubated for 5 min at room temperature and another 5 min at 95 °C. The lysate suspension containing the viral RNA was separated using a magnet rack (MagneSphere^®^ Technology Magnetic Separation Stands, Promega) in fresh, sterile 0.5 mL tubes. The purified lysate suspension was immediately subjected to the RNA amplification step or stored at −80 °C until further analysis. This process for concentration was carried out in accordance with Ceres Nanosciences’ APP-034 (Figure 1).

However, in the PEG-concentrated sample, the PEG suspension in phosphate buffer was treated to RNA extraction according to the predefined methods using the QIAamp Viral RNA Mini Kit (Qiagen, Hilden, Germany), and the purified RNA elute was subjected to the RNA amplification step or stored at −80 °C until further analysis.

### 2.5. RNA Amplification and Detection

In the present study, for rapid amplification of lysate samples from Nanotrap particles or pure RNA extracts from PEG for viral diagnosis in wastewater, we established the QPsor technique using highly sensitive and improved isothermal nucleic acid amplification, RICCA (RNA Isothermal Co-assisted and Coupled Amplification) [46]. Concurrently, SARS-CoV-2 RNA in a sample was independently quantified by conventional RT-qPCR, using QuantiStudio 5 (Thermo Scientific (Waltham, MA, USA)). Pepper mottle mosaic virus (PMMoV) was also quantified in a sample before and after each concentration step in order to validate the detection efficiency. Laboratory-free quantification of viral RNA was also demonstrated by adopting amplification through the PicoGene PCR1100 (Nippon Sheet Glass, Tokyo, Japan), a microfluidic mobile qPCR [47].

The RNA Isothermal Co-assisted and Coupled Amplification reaction (RICCA) is a straightforward one-pot reaction for the detection of low-copy-number RNA viruses in samples without lab processing. The assay was performed using the substrate, primer, and enzyme stock reagents as described by Biyani et al. 2021. The reaction mixture was prepared by mixing 10 μL of substrate stock solution, 10 μL of primer stock solution, and 5 μL of RNA template (lysate suspension/eluted RNA) in a 500 μL Eppendorf tube. After adding 5 μL of enzyme stock solution, the reaction was incubated at 41 °C for 30 min [46]. The CDC-N primer set (Eurofins Genomics, Tokyo, Japan) was used to amplify the SARS-CoV-2 (MN908947.3) template sequence origin 29,195–29,358. Similarly, for PMMoV (M81413.1), PMMV-P1 gene was targeted at sequence origin 1872–2075 (Table 1).

In the context of the RICCA amplification cycle evaluation study, the amplified products were subjected to analysis using a 1-inch 6% denatured polyacrylamide gel and stained with SYBR Gold. Later, RNA amplicons with a length of 164 nucleotides for SARS-CoV-2 were analyzed by allowing them to flow via capillary action on the sample pad of a lateral flow device strip. Prior to the capillary flow of the biotinylated RNA product, the LF pad was hybridized with a digoxin-labeled DNA probe and complexed with a gold-labeled anti-digoxin antibody. This hybridization product was identified and complexed in the form of an immune complex by the biotin ligand of the biotinylated RNA on a lateral flow strip and exposed as a positive test line (T). In the experiment, 25 μL of RNA amplification product was added to 70 μL of detection mix (mix of 1 M KCl, nuclease-free water, and targeted probe nucleotide), incubated at 60 °C, and permitted to flow on the LF test strip at room temperature. The results were read after 5 min.

The microfluidic mobile qPCR assay was carried out using an iCAT direct master mix (iCAT Co., Osaka, Japan) and a microfluidic mobile PCR device (PicoGene^®^ PCR1100, NSG, Niigata, Japan) to identify SARS-CoV-2 in the test wastewater samples in a lab-free real-time PCR quantification direction. To design the experiment, we used the CDC-N gene primers and the readily available TaqMan probe sets accordingly. A microfluidic chip was loaded with a 16 μL mixture containing iCAT direct master mix (14 μL) and test sample (3 μL). The chip was placed into the device and was operated for 50 cycles at the following temperatures and times: 50 °C for 150 s, 95 °C for 15 s, 95 °C for 3.5 s, and 63 °C for 8 s [51]. Excluding the control study, all field trial experiments were conducted one time. The mean value was calculated for the control experiment.

## 3. Results and Discussion

### 3.1. Effectiveness of Nanotrap Particles for Virus Concentration in Wastewater

The chemical and biological complexity of wastewater may reduce the viral yields or the repeatability of virus recovery. In addition, infected human participants can discharge variable titers of SARS-CoV-2 in their feces, which may be extremely diluted in wastewater or are typically present in low concentrations, particularly when case numbers are low with communities [52]. Therefore, a reliable, quick, and real-time approach was developed for SARS-CoV-2 detection in wastewater. As a proof-of-concept study, we compared the virus capture and concentration efficiencies of two approaches: (a) Nanotrap microbiome particles (NTPs, this study) and (b) Polyethylene glycol 8000 precipitations (PEG, conventional method). The results were evaluated by conventional RT-PCR using the Quant iStudio5 real-time PCR machine. The results are shown in Table 2. The findings of the investigation reveal that the targeted gene was positive for all the wastewater samples (SARS-CoV-2 or PMMoV) concentrated using both the NTP and the PEG techniques. For the wastewater samples spiked with SARS-CoV-2 viruses, the Ct values obtained using Nanotrap microbiome particles and Polyethylene glycol 8000 precipitations were Ct 29–36 and Ct 26–33, respectively (Table 2), which revealed a comparable and satisfactory recovery efficiency using NTPs.

However, the recovery efficiency of pepper mottle mosaic viruses (PMMoVs) from raw wastewater samples was significantly less for NTPs, which might be because a binding cross-competition hindrance between types of viruses and NTPs, or NTPs are more specifically optimized for SARS-CoV-2 and enveloped viruses only and less optimized to capture PMMoVs. To improve efficacy, future developments might involve modifying the properties of NTPs to acquire multiplex viral binding capabilities.

The results for PEG precipitation are in close agreement with findings from previous investigations [34,53]. For the control sample in this study, the Nanotrap concentration in a normal saline medium showed better efficiency for recovery than PEG extraction (Supplementary Table S1). It might be because there was a lack of relative inhibitors in the background of wastewater samples that may cause competition among particles to capture the virus. In addition, the results for Nanotrap concentration were also found to be affected by the non-uniform volume of the Nanotrap microbiome particles or a random loss during handling in wastewater. Furthermore, the findings of the conventional quant istudio5 PRR machine demonstrated that the Nanotrap concentration method has a higher capability for collecting viruses in near-sensitivity proportion to the PEG technique. The findings of the investigation reveal that the Nanotrap microbiome particles effectively captured the virus to concentrate viral particles of SARS-CoV-2, present in wastewater, agreeing with previous studies [2,37]. Additionally, the use of a cellulose–acetate membrane-attached syringe filtering process, followed by a volume up from 10 to 30 mL, is also indicative of significant viral concentrations in wastewater samples via the use of a Nanotrap particle. However, the likelihood of virus adsorption was insignificant because of the membrane’s intrinsically limited capacity to bind proteins. Despite this, it was lowered by using a cellulose–acetate membrane that had already been treated with a protein-based material. In addition, the Nanotrap’s potential as a matrix was strongly compatible with the outcome of the PEG procedure. Later, we evaluated the direct heat treatment for Nanotrap particles and the Qiagen RNA extraction kit technique of the PEG concentration to extract SARS-CoV-2 RNA from wastewater samples, followed by RT-qPCR detection and found improved outcomes.

### 3.2. Evaluation of the RICCA Amplification for SARS-CoV-2 Virus and PMMoV

To examine the applicability of RICCA for virus detection in wastewater, we compared the sensitivity and specificity of RNA-specific amplification used to amplify the SARS-CoV-2 virus and the readily available PMMoV (control).

**Figure 2:**
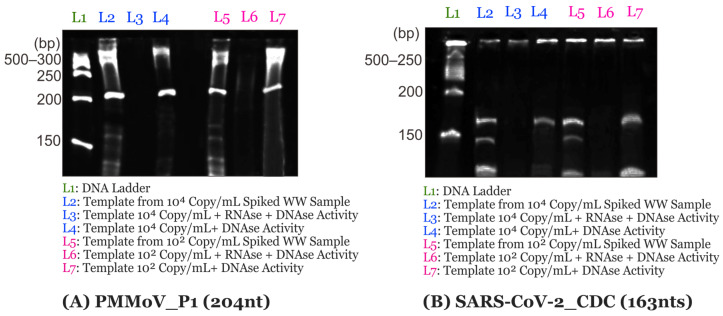
Evaluation of the specificity of RNA-specific amplification of SARS-CoV-2 virus and PMMoV.

The results of polyacrylamide gel electrophoresis in Figure 2 show successful amplifications of RNA amplicons of 204 nts for the PMMoV-P1 gene and 164 nts for the SARS-CoV-2_CDC gene at both spiked concentrations, i.e., 10,000 or 100 copies/mL. The results indicate that the RICCA has a high potential to specifically detect RNA viruses. The produced RNA-specific amplicons were further validated by postreaction treatment of the amplified product with DNase or RNase, which revealed the presence of a band of sense RNA amplification, indicating a clear amplification of the RNA target gene.

### 3.3. Proof-of-Concept Demonstration for QPsor Sensing System

We developed the “QPsor” (Quick Poop Sensor) by directly concentrating spiked viruses in wastewater using Nanotrap microbiome particles followed by directly amplifying the RNA of viruses using the RICCA assay. We spiked 10^4^, 10^3^, and 10^2^ viral copies in wastewater samples to evaluate the sensitivity of the QPsor sensing system.

**Figure 3:**
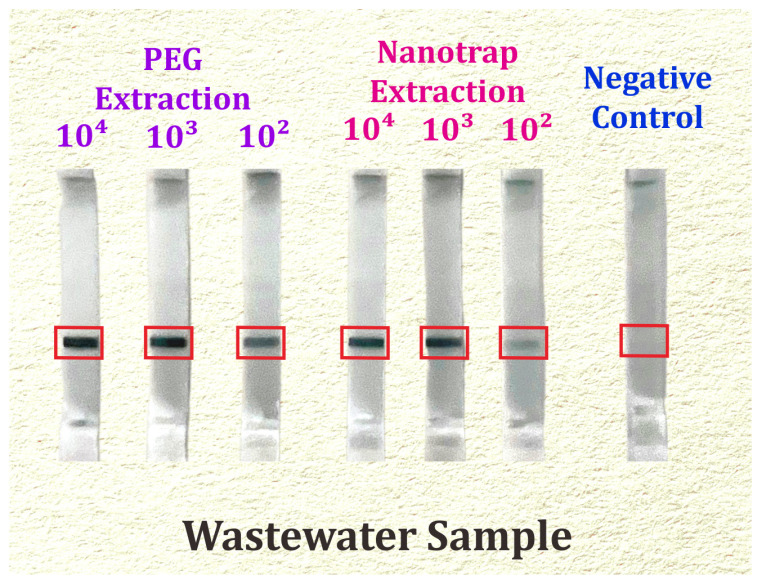
Lab-free detection of SARS-CoV-2 by concentrated lysate/RNA elute-to-RICCA’s RNA amplification-to-LF assay for wastewater samples. Test line results are highlighted by red box.

As shown in the results in Figure 3, a positive test response led to the appearance of a band (test line) within three minutes that gave a dark to faint line band width for RNA amplicons of 164 nts at concentrations as low as 100 copies/mL of the SARS-CoV-2 virus, demonstrating the test’s superior sensitivity for both Nanotrap microbiome particles; and PEG 8000 precipitation. The results indicate that the Nanotrap concentration assay displayed a detection limit of 100 copies/L, which was near-identical to the PEG concentration test. In the negative control, the absence of a positive target band was observed in the reaction without a seeded sample.

### 3.4. QPsor’s Underlying Application for Remote Monitoring of On-Site Wastewater Treatment Plants

**Figure 4:**
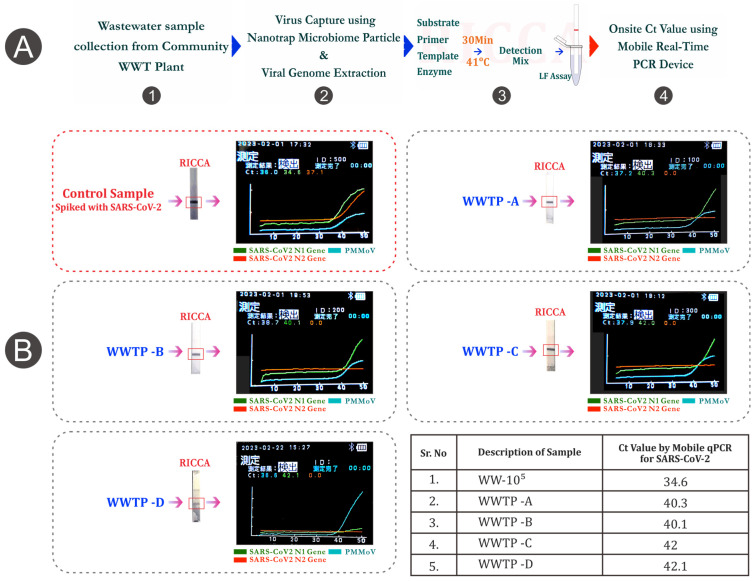
Systematic layout for QPsor system demonstration in laboratory-free settings and viral amplification results for wastewater from multiple wastewater treatment centers in Ishikawa prefecture, Japan. (**A**) layout for workflow and (**B**) results for wastewater samples. Test lines are highlighted by red box.

Next, we demonstrated the field application of the QPsor system by introducing the ‘sample-to-RNA amplification-to-LF assay’ protocol for remotely monitoring the existence of viruses on-site and within 60 min directly from the raw samples at wastewater treatment plants. Four different wastewater treatment plants in Ishikawa prefecture, Japan were studied in this work. As shown in Figure 4, raw samples from all four wastewater treatment plants (WWTP-A, B, C, and D) tested positive for SARS-CoV-2. For comparative evaluation, we used a piece of portable qPCR equipment, the PicoGene PCR 1100, and successfully demonstrated the capability of on-site monitoring and robust practical application in the field of the QPsor system (Figure 4).

In the present study, the Ct value for the N1 gene using portable qPCR in the spiked wastewater control sample (10^5^) was found to be Ct 34.6. Among the four raw samples tested by the PicoGene1100, we observed a very low Ct range of 40–42 for the SARS-CoV-2 virus. The results were cross-evaluated with the conventional PEG and qPCR methods, and were near-consistent (Ct range of 37–40, see Supplementary Table S2). The PMMoV was also detected in all four wastewater samples using NTPs and portable qPCR, establishing a multiplex test of viruses for remote monitoring, with a Ct value ranging from 36 to 38 cycles (Figure 4), which was again consistent with our previous observation using the conventional PEG and qPCR methods (Ct range of 27–28, see Supplementary Table S3). It reveals that the occurrence of multiple viruses in wastewater generates competition between Nanotrap particles to capture identical viruses due to the colloidal nature of wastewater. The findings also exhibit that competition between multiple viruses also weakens the signal obtained by NTPs, which can be further improved by optimizing the protocol for NTP concentration.

Finally, we confirmed that QPsor can be a viable replacement for a time-consuming and expensive qPCR system because it can be used in the field with low-resource settings and yields results within 60 min. The mobile RT-qPCR data also show that the Nanotrap concentration procedure is preferable to the PEG concentration in terms of rapidly capturing viruses within a few minutes and without lab processing. Although the discrepancies in the present study could be attributed to differences in the functionality and principles of the instruments used and the efficiency of the RT-PCR reagents, these explanations are far from conclusive.

## 4. Conclusions

In this study, we developed a field-deployable system, ‘Quick Poop Sensor (QPsor)’ for wastewater surveillance. The QPsor system can handle the chemical or biological complexity of wastewater and detect target viruses, SARS-CoV-2 and PMMoV in this study, without laboratory-based sample processing. Typically, the QPsor test requires 10 min for virus concentration, 5 min for the viral RNA extraction, 15–30 min for the viral RNA amplification, and 3–5 min for viral RNA detection. Therefore, the burden of wastewater sample transport and virus recovery can be managed by enabling rapid multiplexed monitoring for community service users in low-resource settings within 60 min using the QPsor system. We detected SARS-CoV-2 in effluent samples collected from wastewater treatment plants with Ct values ranging from 34.6 to 42.1. The results demonstrate that the QPsor system has a substantial capacity to detect RNA viruses and can be applied to remote sites and thus can be a significant game changer for early-stage management strategies in combating a viral pandemic. It serves as a promising monitoring system for individuals living in remote communities and will therefore be an accessible and affordable complement to traditional types of wastewater surveillance.

## 5. Patents

BioSeeds Corporation has filed the patent application related to this technology with M.B. as the inventor. The other authors declare no competing interests.

## Figures and Tables

**Table 1:** Sequences of primers/probes for RICCA amplification for COVID-19 and PMMoV.

Primers/Probes	Sequence (5′–3′)
2019-nCoV_N1-CDC_F	GTTGTTCGTTCTATGAAGAC
T7-2019-nCoV_N1-CDC-R	AATTCTAATACGACTCACTATAGGGAGATCTGGTTACTGCCAGTTGAATCTG
5DIG-CDC-P1	[Digoxigenir] CGTTCTATGAAGACTTTTTAGAGTATCATG
3-BIOTIN-CDC-P2	CCAAAATCAGCGAAATGCACCCCGCATTAC [Biotin]
PMMV-FP1	AAATGAGAGTGGTTTGACCTT
PMMV-T7-RP1	AATTCTAATACGACTCACTATAGGGAGAAACTCATCGGACACTGTG

**Table 2:** Comparison of viral RNA copy number (Ct value for N1 gene) determined by conventional real-time RT-PCR for samples (lysate/elute) concentrated by Nanotrap microbiome particles and PEG 8000 precipitation method.

S. No.	Sample Types	Spiked/Real Viruses (Copies/mL)	Ct Values		Observed Concentration (Copies/mL)		Recovery Efficiency	
			PEG	NTPs	PEG	NTPs	PEG	NTPs
1	CoV-2_Low	100	33.20 ± 0.02	36.45 ± 0.30	2.1 × 10^2^	1.5 × 10^2^	206%	151%
2	CoV-2_High	10,000	26.53 ± 0.10	29.26 ± 0.14	1.7 × 10^4^	2.0 × 10^4^	170%	196%
3	PMMoV_Low	1.6 × 10^6^	28.3 ± 0.24	39.7 ± 0.00	3.1 × 10^5^	558	19%	0.035%
4	PMMoV_High	2.0 × 10^6^	27.9 ± 0.04	>40	4.5 × 10^5^	ND	23%	ND

PEG: Polyethylene glycol; NTPs: Nanotrap microbiome particles; Ct: Cycle threshold; ND: Not detectable.

## Data Availability

Data are contained within the article.

## Supplementary Materials

The following supporting information can be downloaded at: https://www.mdpi.com/article/10.3390/bios14030115/s1, Table S1: Comparison of viral RNA copy number (Ct value for N1 gene) determined by conventional Real-time RT-PCR for normal saline samples (lysate/elute) concentrated by Nanotrap microbiome particles and PEG 8000 precipitation method; Table S2: Viral RNA copy number (Ct value for the N1 gene) determined by conventional real-time RT-PCR for studied wastewater treatment plant samples (elute) concentrated by the PEG 8000 precipitation method; Table S3: Viral RNA copy number (Ct value for the PMMoV-P1 gene) determined by conventional real-time RT-PCR for studied wastewater treatment plant samples (elute) concentrated by the PEG 8000 precipitation method.

## Abbreviations

AVE	QIAamp viral elution buffer
Ct	Cycle threshold
KCl	Potassium chloride
LF pad	Lateral flow strip pad
NMVPs	Nanotrap magnetic virus particles
NSG	Nippon sheet glass
NTPs	Nanotrap microbiome particles
NaCl	Sodium chloride
PBS	Phosphate-buffered saline
PCR	Polymerase chain reaction
PEG	Polyethylene glycol
PMMoV	Pepper mottle mosaic virus
QPsor	Quick Poop Sensor
RICCA	RNA Isothermal Co-Assisted and Coupled Amplification
RNA	Ribonucleic acid
RT-qPCR	Reverse transcription-quantitative polymerase chain reaction
SARS-CoV-2	Severe acute respiratory syndrome coronavirus 2
WBE	Wastewater-based epidemiology
WWTP	Wastewater treatment plant
qPCR	Quantitative polymerase chain reaction
