# Supplementary material for: Real-Time On-Site Monitoring of Viruses in Wastewater Using Nanotrap® Particles and RICCA Technologies
Source: Biosensors (Basel). 2024 Feb 21;14(3):115. doi: 10.3390/bios14030115 (PMC10967975; doi:10.3390/bios14030115)
Supplement: Supplementary file 1 [file biosensors-14-00115-s001.zip › biosensors-2849868-supplementary.pdf]

Real-Time On-site Monitoring of Viruses in Wastewater using Nanotrap® Particles and RICCA Technologies

Vishnu Sharma[1], Hitomi Takamura[2], Manish Biyani[1*], Ryo Honda[2]

Supplementary Data Sheet

Table-S1: Comparison of viral RNA copy number (Ct value for N1 gene) determined by conventional Real-time RT-PCR for normal saline samples (lysate/ elute) concentrated by Nanotrap microbiome particles and PEG 8000 precipitation method.

Sr. No.	Samples type	Spiked/real viruses (Copies/mL)	Ct values		Observed concentration (copies/ml)		Recovery efficiency	
			PEG	NTP	PEG	NTP	PEG	NTP
1	CoV-2_Low	100	35.8±0.34	25.3±0.04	4.0.E+01	2.9.E+05	40%	290523%
2	CoV-2_High	10000	26.3±0.08	23.5±0.02	1.9.E+04	9.8.E+05	188%	9791%

Table-S2: Viral RNA copy number (Ct value for the N1 gene) determined by conventional real-time RT-PCR for studied wastewater treatment plant samples (elute) concentrated by the PEG 8000 precipitation method.

PEG precipitation

Samples	Spiked SARS-CoV-2 (copies/mL)	Ct values	Observed concentration of CDCN1 (copies/mL)	Recovery efficiency
WWTP A	Unknown	>40	ND	-
WWTP B	Unknown	37.3±0.86	1.7.E+01	-
WWTP C	Unknown	37.3±1.27	1.0.E+01	-

Table-S3: Viral RNA copy number (Ct value for the PMMoV-P1 gene) determined by conventional real-time RT-PCR for studied wastewater treatment plant samples (elute) concentrated by the PEG 8000 precipitation method.

PEG precipitation

Samples	PMMoV in a raw sample (copies/mL)	Ct values	Observed concentration of PMMoV (copies/mL)	Recovery efficiency
WWTP A	1.5.E+06	27.5±0.12	9.1.E+05	62%
WWTP B	1.7.E+06	28.6±0.17	6.1.E+05	37%
WWTP C	2.1.E+06	28.0±0.06	7.8.E+05	37%
